# Transcriptomics of *Listeria monocytogenes* Treated With Olive Leaf Extract

**DOI:** 10.3389/fmicb.2021.782116

**Published:** 2021-12-23

**Authors:** Yanhong Liu, Ting Fang, Yujuan Suo, Shigang Gao, Gian Marco Baranzoni, Cheryl M. Armstrong

**Affiliations:** ^1^Molecular Characterization of Foodborne Pathogens Research Unit, Eastern Regional Research Center, Agricultural Research Service U.S. Department of Agriculture, Wyndmoor, PA, United States; ^2^College of Food Science Fujian Agriculture and Forestry University, Fuzhou, China; ^3^Institute of Agro-Food Standard and Testing Technology, Shanghai Academy of Agricultural Science, Shanghai, China

**Keywords:** *L. monocytogenes*, transcriptomics, RNA-Seq, olive leaf extract, food safety

## Abstract

*Listeria monocytogenes* is a regulated foodborne pathogen that is known to cause listeriosis, a disease associated with high mortality rates in humans. Olive leaf extract (OLE) has been shown to act as a plant antimicrobial and inhibit the growth of pathogens, such as *L. monocytogenes*, although its mode of action has not been defined. To help identify the cellular mechanisms important for conveying these beneficial traits, RNA-Seq was used to study the transcriptome of *L. monocytogenes* upon exposure to a sublethal level of OLE. Results obtained from cells cultured both with and without OLE at two different time points (3.5-h and 24-h) revealed 661 genes that were differentially expressed. Of the differentially expressed genes (DEGs) identified, transcription was altered for 171 genes in response to the 3.5-h OLE treatment while 490 genes were altered in response to the 24-h OLE treatment. These DEGs included but were not limited to genes encoding for signal transduction, ATP-binding cassette (ABC) transporters, and the phosphotransferase system. Interestingly, several virulence-related genes were downregulated including an ABC transporter permease previously shown to negatively regulate biofilm formation, genes involved in flagella assembly and binding/entry into host cells as well as those regulating acid resistance suggesting that OLE may decrease the virulence potential of *L. monocytogenes*. Furthermore, quantitative reverse-transcription PCR was used to validate the data obtained *via* RNA-Seq. Our study provides insight into the mode of action of OLE treatment against *L. monocytogenes* and may aid in identifying synergetic strategies to inhibit *L. monocytogenes* in food.

## Introduction

Olive trees belong to the Oleaceae family, which is comprised of 30 genera (Grohmann, 1981). Olive trees were historically referred to as the “king of trees” because olive leaf extract (OLE) has been shown to be beneficial to humans since ancient times. The beneficial functional and nutritional effects of OLE include antibacterial, antiviral, antifungal, and antioxidant activities (Bisignano et al., 1999; Markin et al., 2003; Micol et al., 2005; Pereira et al., 2007). In addition, OLE has been shown to support the immune system, provide cardioprotection, lower blood pressure, as well as improve diabetes, cancer, and arthritis (Susalit et al., 2011; de Bock et al., 2013; Cabarkapa et al., 2016; Gouvinhas et al., 2017; Xu et al., 2018; Ruzzolini et al., 2020).

*Listeria monocytogenes* is a Gram-positive bacterium known to cause listeriosis in humans. Listeriosis is a life-threatening disease that usually targets immuno-compromised people including newborns, pregnant women, and the elderly. *L. monocytogenes* is widely distributed in the environment and can be found in food and food processing plants. Outbreaks caused by *L. monocytogenes* are often associated with the consumption of milk and dairy product, vegetables, salad, and ready-to-eat (RTE) meats. Unfortunately, controlling this foodborne pathogen can be difficult since it is known to withstand adverse conditions, such as low pH and low temperatures, and forms biofilms, making it more resistant to typical sanitation measures (Farber and Peterkin, 1991; Marriott and Gravani, 2006; Belessi et al., 2011; Etter et al., 2017).

The control of foodborne pathogens is important in the food industry, and the antimicrobial activities of OLE make it possible to be used as a natural antimicrobial in this setting (Liu et al., 2017). OLE has been shown to have the potential to be used as a food packaging material (Liu et al., 2018). It can also be made into functional foods and used as a food preservative (Thielmann et al., 2017) to enhance food quality and shelf-life. The safety of olive extract as an antioxidant or antimicrobial agent in foods has been assessed (Soni et al., 2006).

A major component of OLE is the polyphenols with oleuropein, verbascoside, luteolin-7-o-glucoside, and luteolin-4-o-glucoside being the main constitutes (Liu et al., 2017). In our previous study, OLE was shown to inhibit the growth of foodborne pathogens including *L. monocytogenes*, *Salmonella* Enteritidis, and *Escherichia coli* O157:H7. Furthermore, OLE has also been shown to reduce the cell motility and biofilm formation in *L. monocytogenes* (Liu et al., 2017, 2018). Polyphenol extract from olive oil also induced damage to the bacterial cell membrane, thus affecting its permeability and resulting in a leakage of cytoplasmic components (Guo et al., 2019). In addition, a systems-based biology approach was used to explore the antimicrobial mechanism of oleuropein, the major component of OLE. It was found that oleuropein not only slowed the growth but also inhibited the activities of several metabolic enzymes (Li et al., 2016).

At the genomic level, a microarray study was used to explore gene expression alterations in *E. coli* K-12 grown at 37°C in the presence of a sublethal dose of polyphenol extracts from olive mill wastewater. It was found that genes related to biofilm formation and cell motility were downregulated. These gene expression data were further confirmed by crystal violet assays in addition to assays conducted for swimming and swarming (Carraro et al., 2014). RNA-Seq technology has been used to replace microarrays for the study of gene expression patterns in bacterial cells and plants. For example, differentially expressed genes (DEGs) in tomatoes have been identified in response to ethylene inhibitor 1-methylcyclopropene (1-MCP) treatment (Bobokalonov et al., 2018). In another study, DEGs in *L. monocytogenes* treated with organic acid (sodium lactate) were identified using RNA-Seq technology (Suo et al., 2018). In this study, RNA-Seq analysis was used to characterize changes to the transcriptome of *L. monocytogenes* post-exposure to OLEs to better understand the effects of OLE on *L. monocytogenes*. This information will assist in the development of new intervention strategies aimed at controlling *L. monocytogenes*.

## Materials and Methods

### OLE Stock Preparation

Capsules containing 500 mg of OLE were purchased from General Nutrition Centers (GNC) health stores and an OLE stock solution (25%) was prepared as described previously (Liu et al., 2017, 2018).

### Treatment of *L. monocytogenes* F2365 Cells With OLE

In this study, *L. monocytogenes* F2365, a strain originally isolated from a Mexican-style soft cheese that was associated with an outbreak of listeriosis in California (Linnan et al., 1988) was utilized. Single colonies of *L. monocytogenes* F2365 were inoculated in 5 ml Brain Heart Infusion (BHI) broth (BD/Difco Lab., Sparks, MD, United States) at 30°C overnight with 200 rpm agitation. 100 μl of bacterial overnight cultures were added to 4.744 ml of BHI with 156 μl of OLE stock (25%), yielding a final concentration of 0.78% of OLE. The bacterial cultures were then incubated at 30°C with 200 rpm agitation for either 3.5 h or 24 h, to represent the log phase and stationary phase of growth, respectively. This temperature was chosen as to not bias the transcriptomic studies by utilizing a temperature (such as 37°C) known to repress motility and flagellin production in *L. monocytogenes* (Peel et al., 1988; Kathariou et al., 1995) since both of these factors are known to be related to *L. monocytogenes* growth in foods and processing environments (Vatanyoopaisarn et al., 2000). As controls, 100 μl of bacterial overnight cultures were added to 4.9 ml of BHI and incubated at 30°C with 200 rpm agitation for either 3.5 h or 24 h. For each sample, 1.5 ml bacterial suspension was placed into 1.5 ml RNase-free tubes and centrifuged at 8,000 rpm at 4°C for 5 min. After discarding the supernatant, the pellets were resuspended in 500 μl RNAlater (Thermo Fisher, Waltham, MA) and stored in a −80°C freezer until further RNA isolation. Three biological samples were prepared for each treatment.

### RNA Isolation, Ribosomal RNA Depletion, Library Construction, and Sequencing

The RNA extraction was performed using the RiboPure – Bacteria kit (Thermo Fisher, Waltham, MA) as per the manufacturer’s instructions with the following modification: bacterial cells were disrupted using a FastPrep-24 5G homogenizer (M.P. Biomedicals LLC, Santa Ana, CA) at maximum setting for 5 min. The integrity of the RNA was analyzed with an Agilent 2100 Bioanalyzer (Agilent Technologies, Wilmington, DE), ensuring that all samples had an RNA integrity number (RIN) over 7. Residual genomic DNA was degraded using the TURBO DNA-free kit (Thermo Fisher, Waltham, MA). Briefly, 50 μl of each sample were mixed with 5 μl 10× buffer and 2 μl of TURBO DNase and incubated at 37°C for 30 min. 5 μl of DNase inactivation reagent were added to all mixes and incubated for 5 min at room temperature. The mixture was centrifuged at 10,000 × *g* for 1.5 min. RNA was cleaned using the RNA Clean & Concentration kit (Zymo Research, Irvine, CA). All the centrifugation steps were performed at 10,000 × *g*, and RNA was eluted in 15 μl twice. DNA-free RNA samples were quantified using the RNA BR Qubit assay kit (Thermo Fisher, Waltham, MA). Removal of the bacterial rRNA was performed using the Ribo-Zero rRNA Removal Kit for Bacteria (Illumina, San Diego, CA) as per the manufacturer’s protocol. A cDNA library was generated using the Ion Total RNA-Seq Kit v2 and equal amounts of extracted RNA as per the manufacturer’s instructions. The cDNA libraries were checked using an Agilent 2100 Bioanalyzer (Agilent Technologies, Wilmington, DE) prior to sequencing on Ion Chef and Ion S5 instruments (Thermo Fisher Scientific) as per the manufacturer’s protocol. All of the RNA-Seq experiments were repeated two or three times.

### Transcriptomic Data Analysis

The raw data quality of the Ion Torrent reads was verified using the fast QC program. Mapping of raw sequence reads was performed using the Subjunc aligner from Subread (Liao et al., 2013) and the genome/gene annotations for *L. monocytogenes* str. 4b F2365 (NCBI accession AE017262; Nelson et al., 2004), with a majority of the reads obtained (~69–88% for all samples) aligning to the genome. Comparisons between the alignment bam files and the gene annotation GTF files were made, and raw counts were generated for individual genes *via* the Feature Counts tool from Subread, resulting in ~12–69% of reads being assigned to genes. The voom method, a component of the R Limma package, was used to normalize raw counts data and for the analysis of differential gene expression (Ritchie et al., 2015). Raw sequence data were deposited into the NCBI BioProject database under BioProject ID number PRJNA382175.

### Identification of DEGs, Sample Grouping, and Function

Relative gene expression levels were estimated using reads per kilobase per million (RPKM) values, and the limma package was used to compute the significance of differential gene expression using raw counts of genes (Smyth, 2004). RPKM values were computed using the extracted raw-read counts from individual genes that had been converted to counts per million (CPM) to normalize for the sequencing depth. The CPM data were subsequently divided by the length of the gene (kb) to normalize for the length of the transcript. Log_2_ Fold Change (Log_2_FC) ≥ 1 and Log_2_FC ≤ −1 were employed as the filter to define genes up- and downregulated by at least two-fold, respectively. Significance differences in gene expression were defined using a value of *p* < 0.01 and False Discovery Rate (FDR) < 0.05.

A multidimensional scaling (MDS) plot allowed the visualization of sample relationships. Functional enrichments of DEGs were conducted by Gene Ontology (GO) and Kyoto Encyclopedia of Genes and Genomes (KEGG) pathways. The significance of the GO term enrichments and KEGG pathways in DEGs was defined using a threshold value of *Q* ≤ 0.05.

### Quantitative Real-Time PCR Assays

Primers based upon the gene sequences were designed using the Primer3 (v. 0.4.0) software.^1^ Primer sequences are listed in Supplementary Table S1. Synthesis of cDNA was performed as previously described (Liu and Ream, 2008) with *spoG* used as an internal control. The Quant Studio 6 Flex real-time PCR system (Thermo Fisher, Foster City, CA) was employed for the quantitative reverse-transcription PCR (qRT-PCR) analysis as previously described (Bobokalonov et al., 2018), and all experiments were performed in triplicate.

### Statistical Analyses

For each experiment, three replicates were used for each treatment, the experiments were repeated two times. Significance was established through Student *t*-tests using values of *p* < 0.05.

## Results and Discussion

### Analysis of Transcriptomics Data and DEGs of *L. monocytogenes* Treated With OLE

Our previous study showed that OLE inhibited *L. monocytogenes* growth with a minimal inhibitory concentration (MIC) of 6.25% in BHI media (Liu et al., 2017). At the sublethal dose of 1/8 × MIC (0.78%), 47% inhibition of *L. monocytogenes* was achieved after a 24-h treatment (Liu et al., 2018). At this sublethal dose, biofilm formation was also inhibited compared to non-treated controls and flagella was lost (Liu et al., 2017). Therefore, this sublethal dose (0.78% OLE) was selected as the treatment to study the resulting transcriptome in *L. monocytogenes*, with cells being grown for 3.5-h (log-phase) and 24-h (stationary phase) time periods. RNA was isolated and subjected to RNA-Seq analysis. Bacterial cells without OLE grown under the same conditions (3.5 h and 24 h) were used as controls.

Changes to the gene expression profile of *L. monocytogenes* treated with a sublethal dose of OLE were also analyzed *via* RNA-Seq using the Ion Torrent platform. As shown in Table 1, the raw sequence reads ranged from 6,820,510 to 10,397,703 with a mean read length from 132 to 159 bp and 69.9–87.8% of the sequence reads mapping to the *L. monocytogenes* F2365 genome (GenBank Accession AE017262). Of the 2,906 total genes predicted in the *L. monocytogenes* F2365 genome, 2,808 genes were expressed.

**Table 1:** Overall quality of RNA-Seq data.

Sample name	Total_Reads	Percentage_Mapped	Mean read length (bp)
BHI_3.5h_1	10,397,703	69.9	159
BHI_3.5h_2	8,734,095	76.9	139
olive_3.5h_1	6,820,510	77.9	136
olive_3.5h_2	8,513,534	80.1	149
BHI_24h_1	7,024,285	87.8	145
BHI_24h_2	9,375,486	83.7	144
BHI_24h_3	7,303,664	86	132
olive_24h_1	9,145,233	87.8	136
olive_24h_2	9,750,564	84.4	147
olive_24h_3	7,451,900	86	145

Multidimensional scaling plots were used to show good separation between samples under different OLE treatments (Supplementary Figure S1). As shown in Supplementary Figure S1, similarities were present among treatment replicates (3.5 h and 24 h) while sample treatments differed from one another (Supplementary Figure S1).

**Figure 1:**
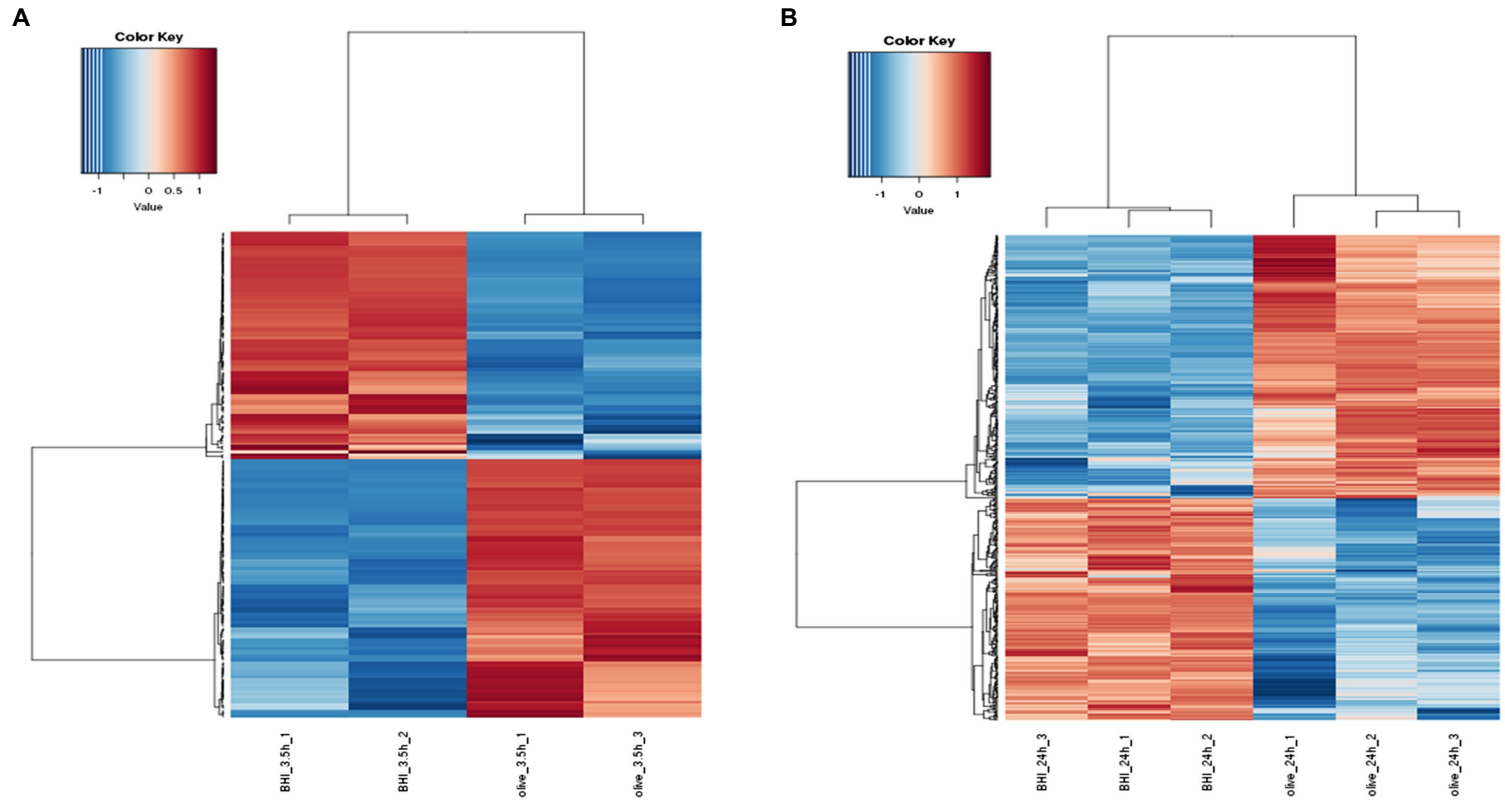
Response of the differentially expressed genes (DEGs) to olive leaf extract (OLE) treatment in *Listeria monocytogenes*. DEGs shown to be upregulated at **(A)** 3.5 h and **(B)** 24 h post-OLE treatment are displayed in red whereas those shown to be downregulated are blue in color.

**Figure 2:**
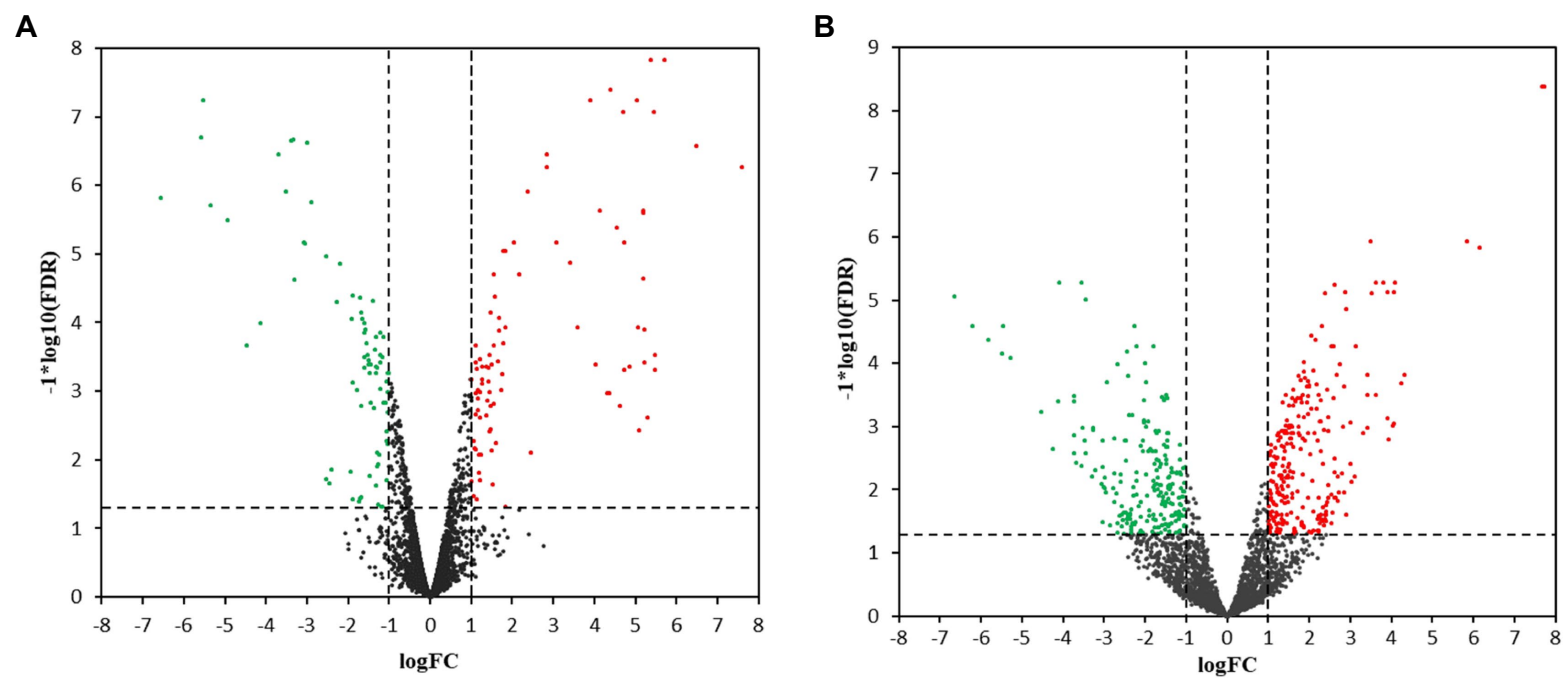
Volcano plots for transcriptional profiles. Transcriptional changes resulting in the upregulation (red dots) or the downregulation (green dots) of genes were identified using scatter plots at **(A)** 3.5 h and **(B)** 24 h post-OLE treatment. Dashed lines represent values of *p* > 0.05 and genes demonstrating no change in expression level are shown by black dots. FDR, False Discovery Rate; FC, Fold Change.

Hierarchical cluster analysis and volcano plots were used to represent variances in DEGs (Figures 1, 2). Of the 2,808 genes analyzed, 171 DEGs were identified in the 3.5-h OLE treatment, with 91 and 80 being up- (Log_2_FC ≥ 1, FDR < 0.05) and downregulated (Log_2_FC ≤ −1, FDG < 0.05), respectively (Supplementary Table S2). 490 DEGs were identified in the 24-h OLE-treated cells, with 266 and 224 up- and downregulated genes, respectively (Supplementary Table S3). Additionally, expression patterns for all DEGs were found to be similar among treatment replicates (Figure 1) while the MDS plot, clustering, and volcano plot analysis showed high reproducibility between treatments for the RNA-Seq experiments.

**Figure 3:**
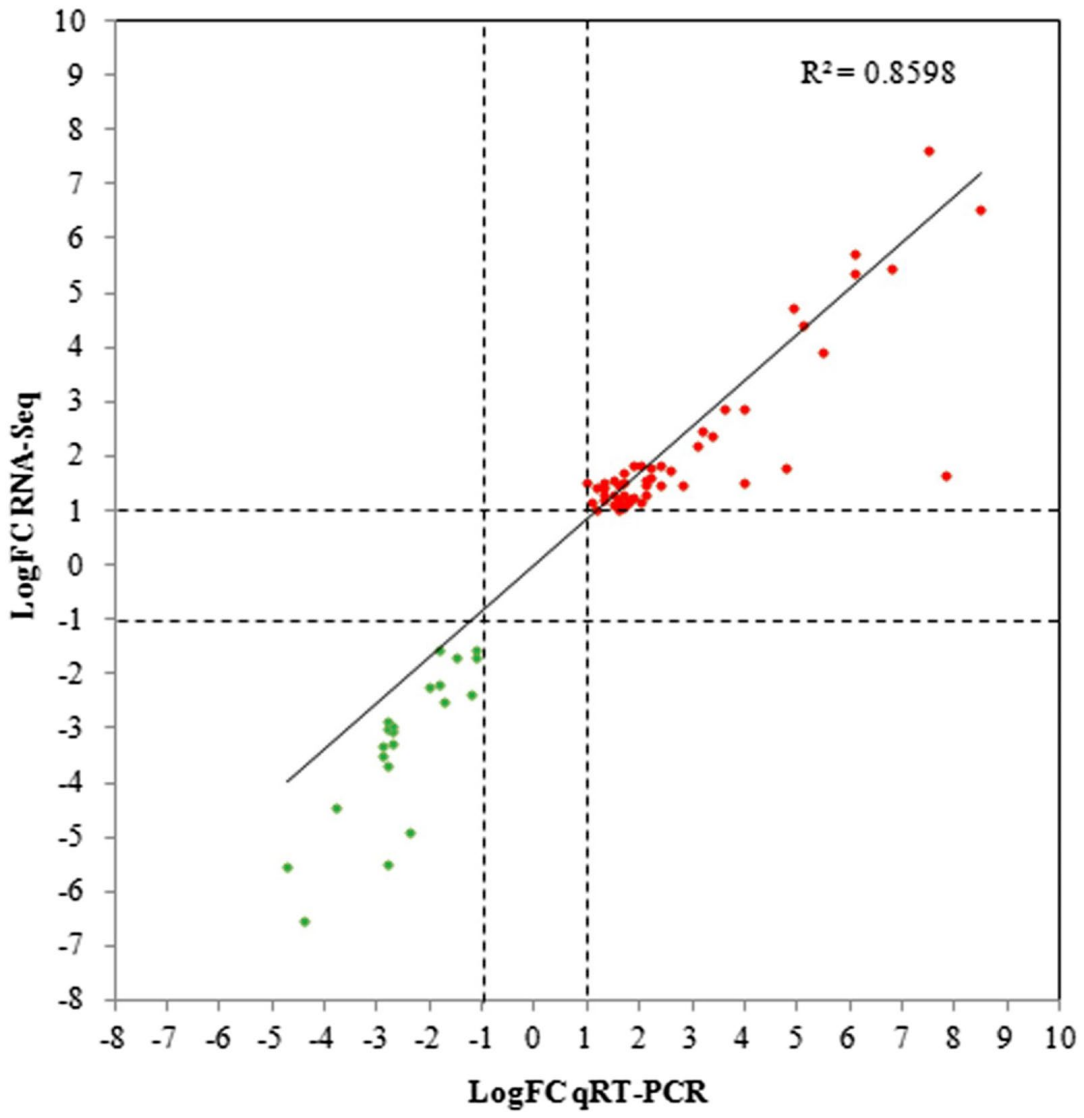
Quantitative reverse-transcription PCR (qRT-PCR) validation for selected DEGs. Linear regression plots of the log Fold Change (logFC) obtained *via* RNA-Seq compared to qRT-PCR for 72 DEGs selected from the 3.5-h OLE treatment. Red dots represent upregulated genes whereas green dots represent downregulated genes.

In addition, qRT-PCR analysis of 72 genes selected from the DEGs in the 3.5-h OLE treatment was conducted to validate the RNA-Seq data. As shown in Figure 3 and Supplementary Table S1, data obtained *via* qRT-PCR correlated well with that obtained *via* RNA-Seq (*R*^2^ = 0.86), indicating a high degree of precision among the data collected. Differences between the log_2_ FC obtained *via* qRT-PCR compared to that obtained *via* RNA-Seq were ≤ 1.0 in 60 of the 72 selected genes and ≤ 2.0 in 66 of the genes analyzed.

### Expression Analysis Using GO and KEGG

**Figure 4:**
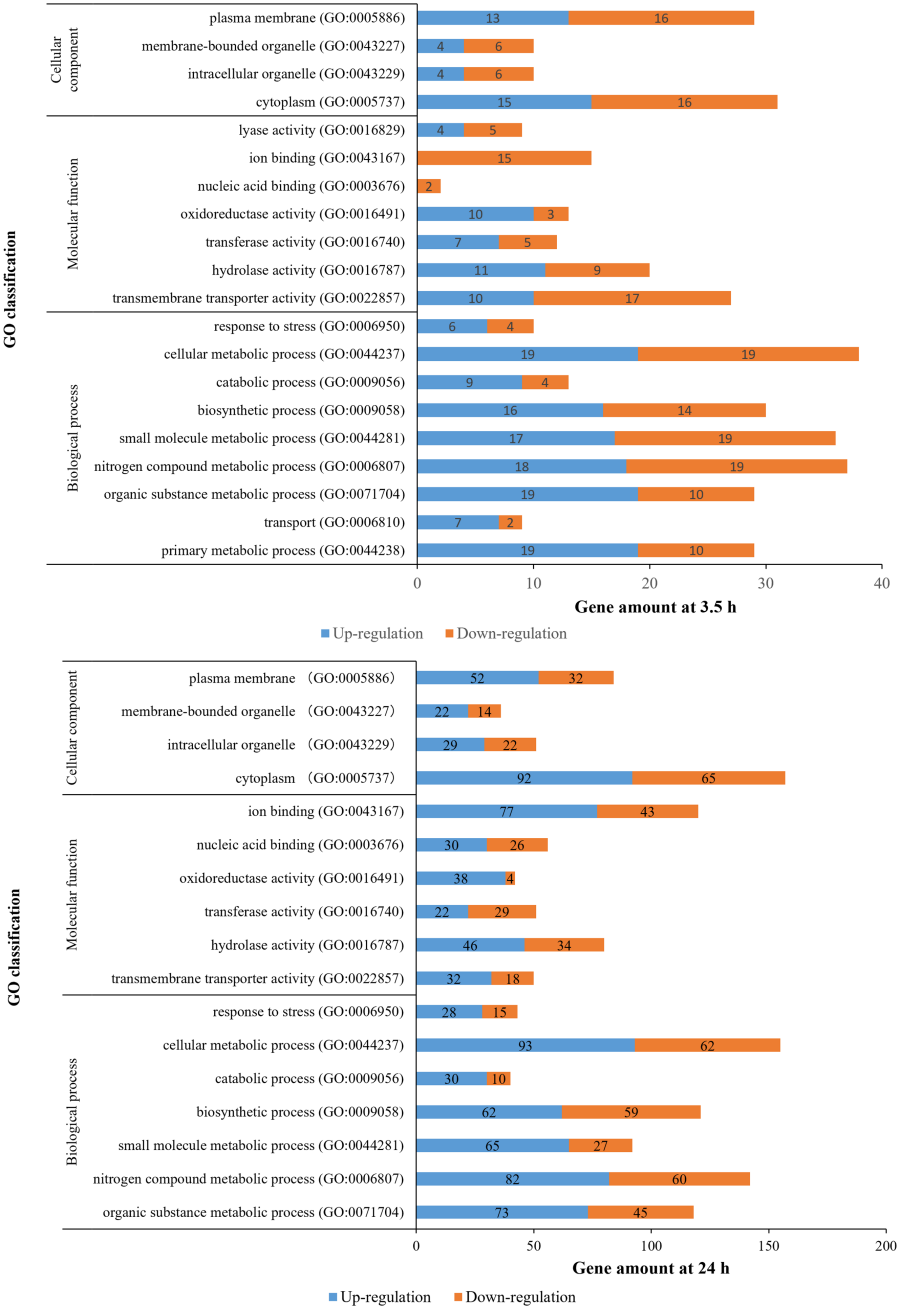
Gene ontology classification of DEGs for OLE treatments in *L. monocytogenes*.

The distribution of the DEGs was studied by GO enrichment analysis for gene function in terms of cellular component (CC), molecular function (MF), and biological process (BP). There were 20 (BP: 9 terms, MF: 7 terms, CC: 4 terms) and 17 GO categories (BP: 7 terms, MF: 6 terms, CC: 4 terms) that were differentially enriched in 3.5-h and 24-h treatments, respectively (Figure 4). For the 3.5-h OLE treatment, the most enriched GO terms were cellular metabolic process (GO:0044237, 38 DEGs), nitrogen compound metabolic process (GO:0006807, 37 DEGs), small molecule metabolic process (GO:0044281, 36 DEGs), plasma membrane (GO:0005886, 29 DEGs), primary metabolic process (GO:0044238, 29 DEGs), and transmembrane transporter activity (GO:0022857, 27 DEGs). For the 24-h OLE treatment, the most enriched GO terms were cytoplasm (GO:0005737, 157 DEGs), cellular metabolic process (GO:0044237, 155 DEGs), nitrogen compound metabolic process (GO:0006807, 142 DEGs), biosynthetic process (GO:0009058, 121 DEGs), ion binding (GO:0043167, 120 DEGs), and organic substance metabolic process (GO:0071704, 118 DEGs). KEGG pathways were also used to align all DEGs to identify major pathways. In 3.5-h and 24-h OLE-treated samples, DEGs were matched in 147 and 565 pathways, respectively. The top enriched pathways in 3.5-h OLE treatment samples were as follows: 01100 Metabolic pathways (21 DEGs), 02010 ABC transporters (12 DEGs), 02020 Two-component system (10 DEGs), 00240 Pyrimidine metabolism (10 DEGs), 01120 Microbial metabolism in diverse environments (7 DEGs), and 01110 Biosynthesis of secondary metabolites (7 DEGs; Supplementary Table S4). The top enriched pathways in 24-h treatment samples were: 01100 Metabolic pathways (93 DEGs), 01110 Biosynthesis of secondary metabolites (41 DEGs), 01120 Microbial metabolism in diverse environments (26 DEGs), 01130 Biosynthesis of antibiotics (32 DEGs; Supplementary Table S5).

### Analysis of DEGs That Were Expressed in Both OLE-Treated (3.5-h and 24-h) Samples

Thirty-seven DEGs were identified in both 3.5-h and 24-h treatments (Supplementary Table S6). These included genes encoding transcriptional regulators (LMOf2365_2566, LMOf2365_2715, and LMOf2365_0343), ABC transporters (LMOf2365_1264, LMOf2365_0637, LMOf2365_2357, and LMOf2365_2147), two-component systems (LMOf2365_0994 and LMOf2365_2662), virulence (LMOf2365_0213), membrane and cell wall related (LMOf2365_2330 and LMOf2365_2212), antimicrobial resistance and transport (LMOf2365_2560), etc. In addition, several genes with unknown function were also regulated. Of the 37 genes, 17 genes were upregulated in both 3.5-h and 24-h OLE-treated samples, 16 genes were downregulated in both OLE-treated samples, whereas 4 genes (*fru*B, *cad*A, *nrd*D, and *nrd*G) were not consistently up- or downregulated across the two timepoints.

### OLE Alters the Expression of Genes Involved in Both Growth and Survival of *L. monocytogenes*

In general, our data not only support the previous findings that OLE can reduce biofilm formation and cell motility (Liu et al., 2017, 2018), virulence, intracellular ATP, DNA and protein synthesis, as well as increase membrane permeability (Guo et al., 2019) but also provide an understanding of the mechanisms behind such actions. For instance, mechanisms for antimicrobial resistance in Gram-positive bacteria are known to include modifications to the cell wall and membrane, and efflux systems (Nawrocki et al., 2014). During our analysis of the transcriptome, changes to the expression of genes involved in the above processes were uncovered, such as those within the *dlt* operon. This four gene operon (*dltA-D*) is involved in cell wall modification, acting to incorporate D-alanine residues into the cell wall-associated lipoteichoic acids, and has been shown to effect both susceptibility to cationic peptides and biofilm formation (Abachin et al., 2002; Alonso et al., 2014). Moreover, inactivation of *dltA* coincided with a decrease in cellular adherence to a human epithelial cell line and a 4-log increase in the LD_50_ in a mouse infection model (Abachin et al., 2002). In our study, all four genes within the *dlt* operon were found to be downregulated 3.5 h post-exposure to OLE, indicating a mode of action for some of the phenotypic effects seen after treatment with OLE.

Our study also revealed that the transcripts of 8 ABC transporters (Liu et al., 2012) were moderately altered 3.5 h after treatment with OLE (6 being upregulated and 2 downregulated) and by 24 h post-treatment, alterations could be seen in 19 ABC transporters (9 being upregulated and 10 downregulated). One transporter of particular importance is that encoding for an ABC transporter permease (LMOf2365_1771), which has been predicted to play a role in biofilm formation through the exportation of a signal inhibiting biofilm formation (Zhu et al., 2008). Based upon our RNA-Seq analysis, transcript levels of this negative regulator were increased (Log_2_FC = 1.61) when *L. monocytogenes* was grown in the presence of OLE for 24 h; thus, providing an additional mechanism by which OLE may inhibit biofilm formation. Interestingly, homologues of LMOf2365_1771 with a high degree of sequence identity (>95%) were found in other *Listeria* genomes, suggesting that biofilm reduction by OLE *via* this mechanism may be widespread among the *Listeria* species.

Other transport systems affected by the addition of OLE included the phosphotransferase transport system (PTS), with the expression of 4 genes (LMOf2365_2305, LMOf2365_2664, LMOf2365_2665, and LMOf2365_2762), being differentially regulated at the 3.5-h treatment period (Log_2_FC = 5.71, −1.36, −1.47, and 1.13, respectively). At 24 h post-treatment, 9 PTS-related genes (LMOf2365_0030, LMOf2365_0113, LMOf2365_0572, LMOf2365_0922, LMOf2365_1272, LMOf2365_2128, LMOf2365_2129, LMOf2365_2130, and LMOf2365_2292) were found to be differentially regulated; 7 of which were found to be downregulated in the presence of OLE while LMOf2365_2129 and LMOf2365_0572 were both upregulated. It is likely that alterations in the transcript level of these metabolic-related genes are related to the reduction in intracellular ATP previously reported (Guo et al., 2019).

Interestingly, of the 4 genes that were differentially regulated at both timepoints yet not consistently up- or downregulated across these two timepoints, one encoded for the anaerobic ribonuclease reductase *nrdD* (LMOf2365_0299) and another for its activating protein *nrdG* (LMOf2365_0300). Ribonuclease reductases are part of the only known biochemical pathway for the *de novo* synthesis of deoxyribonucleotides and produce all four deoxyribonucleotides needed for DNA synthesis (Jordan and Reichard, 1998). Since both *nrdD* and *nrdG* showed decreased expression during log phase in the presence of OLE but increased expression during stationary phase compared to cells grown in BHI alone, we suspect that these deviations would ultimately lead to a negative impact on the growth of *L. monocytogenes* under anaerobic conditions. It is also likely that additional phenotypes related to alterations in the natural expression patterns of other essential genes by OLE may be observable under certain conditions. However, additional studies outside the scope of this work would need to be conducted to confirm these hypotheses.

Although our plaque assay indicated that OLE did not outwardly affect the virulence of *L. monocytogenes* at the sublethal level used in this study (data not shown), transcription of several major virulence genes was altered by the treatment. At 24 h post-treatment with OLE, *inlA* (LMOf2365_1772, a surface protein that mediates entry of the bacterium into host cells) was elevated while *ami* (LMOf2365_1540, an autolytic amidase that facilitates binding) was reduced (Log_2_FC = 1.50 and −1.42, respectively). Furthermore, a gene important for flagella assembly (LMOf2365_0726) was moderately reduced 3.5 h post-treatment, which correlates with the reduced motility and the absence of flagella previously observed for *L. monocytogenes* in the presence of OLE (Liu et al., 2017). The fact that at 24 h post-treatment no difference was seen between the OLE-treated and non-treated controls was as expected since this gene is typically downregulated during stationary phase. Among the genes consistently downregulated at both the 3.5-h and 24-h treatment periods (Log_2_FC = −1.44 and −1.74, respectively) was *hly* (LMOf2365_0213), which encodes the pore-forming toxin listeriolysin O and is known to mediate the escape of the bacterium from the vacuole (Gaillard et al., 1987). Listeriolysin O has also been shown to play a role in the internalization of the bacterium by nonphagocytic cells (Phelps et al., 2018), signifying multiple methods by which the downregulation of *hly* could affect the virulence potential of *L. monocytogenes*. The ability of *L. monocytogenes* to survive in the acidic environment found in the stomach has been directly linked to glutamate-mediated acid tolerance orchestrated *via* glutamate decarboxylase activity (GAD; Cotter et al., 2001). This system works by converting glutamate into gamma-aminobutyrate, which consumes an intracellular proton in the process and effectively lowers the proton concentration within the cell. In the presence of OLE, transcripts related to GAD appear reduced (LMOf2365_2333, LMOf2365_2334, and LMOf2365_2405), which is indicative of acid sensitivity.

Lastly, several transcriptional regulators appear to be affected by the addition of OLE. These include a putative transcriptional activator (LMOf2365_0343) and three regulators within the MerR family of transcriptional regulators, two of which were found to be highly upregulated at both 3.5 and 24 h when in the presence of OLE (LMOf2365_2566 and LMOf2365_2715) and one (LMOf2365_0100) that was found to be slightly upregulated only at the 24-h timepoint. Although specific actions for these regulators have not been defined, members of the MerR family are known to respond to environmental stimuli including oxidative stress, heavy metals, and antibiotics (Brown et al., 2003). Additionally, a mutant strain of *L. monocytogenes* with decreased biofilm formation was shown to have an insertion in a transcriptional regulator belonging to the MerR family of regulators (Huang et al., 2012).

### Plant-Based Compounds With Modes of Action Similar to OLE

It is worth noting that other plant extracts have been shown to affect the virulence of *L. monocytogenes* using modes of action similar to that of OLE. Included among these are stevia, a well-known natural sweetener derived from *Stevia rebaudiana*, which has been shown to inhibit the growth of *L. monocytogenes* (Abou-Arab and Abu-Salem, 2010). This sugar substitute appears to affect the hemolytic activity of listeriolysin O (Sansano et al., 2017), which is encoded for by *hly* and was one of the genes consistently downregulated at both treatment periods in the present study with OLE. Pomegranate rind extract, which can reduce the ability of *L. monocytogenes* to adhere to and invade cells, was also found to decrease the level of *hly*, *prfA*, and *inlA* produced (Xu et al., 2015). In addition to those already mentioned, Carvacrol and Thymol (oregano oil extracts obtained from *Origanum glandulosum*), and Trans-cinnamaldehyde (a bark extract from *Cinnamomum zeylandicum*) were also shown to impact attachment and invasion by affectively decreasing *prfA* and *hly* (Upadhyay et al., 2012). Moreover, *hly* was not the only virulence gene affected by both OLE and these three compounds, with *lmo1647*, *lmo1076*, and *iap* being altered as well.

This is by no means an exhaustive list of the plant-based compounds known to inhibit *L. monocytogenes*. As investigations into plant-based compounds continue, not only will new compounds be identified but the mechanisms shared among them will also be revealed. This novel insight may provide us with alternatives to some of the synthetic chemicals currently used as antimicrobials.

### Summary

In summary, RNA-Seq was used to analyze the transcriptome of *L. monocytogenes* treated with a sublethal dose of OLE. Some DEGs identified were constituents of molecular pathways including those related to transport (ABC transporters and PTS), cell motility, attachment, acid resistance, and biofilm formation, which correlated with phenotypes previously described for cells grown in the presence of OLE. Because of the benefits associated with human health and the ability to inhibit the growth of bacterial pathogens that OLE imparts, OLE has the potential to be used as a safe antimicrobial with good nutritional value in food. Therefore, knowledge of the mechanisms important for conveying these beneficial traits may assist with future intervention strategies designed to control *L. monocytogenes* in foods. Further investigations aimed at defining OLE levels that reduce food product contamination without altering factors, such as the taste and texture, of a product are also warranted.

## Data Availability Statement

The datasets presented in this study can be found in online repositories. The names of the repository/repositories and accession number(s) can be found in the article/Supplementary Material.

## Author Contributions

YL and GB contributed to study conception and design. YL, TF, and GB performed the experimentation and data acquisition. YS and SG analyzed and interpretated the data. YL drafted the manuscript. YL and CA carried out critical revisions. The final manuscript was both read and approved by all authors.

## Funding

This research was supported by the U.S. Department of Agriculture, Agricultural Research Service.

## Conflict of Interest

The authors declare that the research was conducted in the absence of any commercial or financial relationships that could be construed as a potential conflict of interest.

## Publisher’s Note

All claims expressed in this article are solely those of the authors and do not necessarily represent those of their affiliated organizations, or those of the publisher, the editors and the reviewers. Any product that may be evaluated in this article, or claim that may be made by its manufacturer, is not guaranteed or endorsed by the publisher.

## Footnotes

^1^ http://bioinfo.ut.ee/primer3-0.4.0/
