# Supplementary figures and images for: Transcriptomics of Listeria monocytogenes Treated With Olive Leaf Extract
Source: Front Microbiol. 2021 Dec 23;12:782116. doi: 10.3389/fmicb.2021.782116 (PMC8740304; doi:10.3389/fmicb.2021.782116)

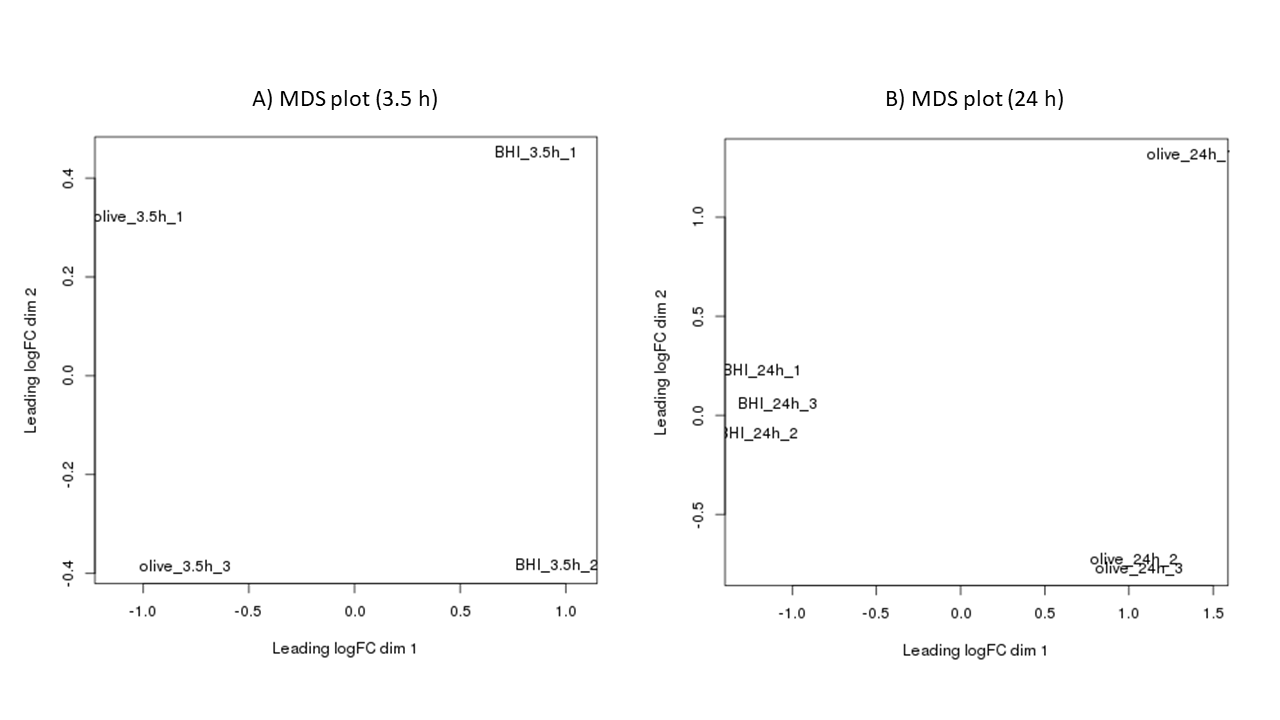

Supplement: Supplementary file 2 [file Image_1.TIF]
